# The Effect of Menthol Mouth Rinsing and Fluid Temperature on Male Cycling Performance in Thermoneutral Conditions

**DOI:** 10.3390/nu16071016

**Published:** 2024-03-30

**Authors:** Erica H. Gavel, Kierstyn V. Hawke, Heather M. Logan-Sprenger

**Affiliations:** 1 Faculty of Science, Ontario Tech University, 2000 Simcoe St N, Oshawa, ON L1G 0C5, Canada; heather.sprenger@ontariotechu.ca; 2 Departments of Family Medicine and Physical Medicine and Rehabilitation, University of Michigan, Ann Arbor, MI 48109, USA; 3 Tanenbaum Institute for Science in Sport, University of Toronto, Toronto, ON M5T 1R8, Canada; 4 Faculty of Health Science, Ontario Tech University, Oshawa, ON L1G 0C5, Canada; kierstyn.hawke@ontariotechu.net

**Keywords:** menthol, swilling, performance, cycling, thermoneutral environment

## Abstract

Purpose: The purpose of this study was to determine the effect of a menthol (MEN) mouth rinse (MR) on cycling time trial (TT) performance in thermoneutral conditions and to explore the impact of fluid temperature (cold water [CW] or thermoneutral water [TNW]) on MEN’s effect on performance. Methods: Twelve trained male cyclists (*V*O_2 peak_, 61.4 ± 12.1 mL/kg/min) completed a cycling TT in thermoneutral conditions (21 ± 0.2 °C, 40 ± 0.6% relative humidity) with four different mouth rinses: (1) MEN + CW; (2) MEN + TNW; (3) CW; and (4) TNW. The time to complete the TT and the power output (W) were recorded. The ratings of perceived exertion (RPE, Borg 6-20), thermal sensation (TS), and thermal comfort (TC) were recorded prior to and throughout the TT. The core body temperature (Tc) and heart rate (HR) were recorded throughout. Results: The TT duration was not significantly different between trials (MEN + TNW: 38:11 ± 12:48, MEN + CW: 37:21 ± 13:00, CW: 38:12 ± 13:54, TNW: 36:06 ± 14:12 mins:secs, *p* < 0.05). The mean trial power output did not significantly differ between conditions (>0.05). The Tc, HR, RPE, TS, and TC were not significantly different between trials (*p >* 0.05). Conclusion: The results suggest that a MEN MR with either CW or TNW does not significantly improve cycling TT performance in trained male cyclists compared to a CW or TNW MR in thermoneutral conditions.

## 1. Introduction

Menthol (MEN), an organic compound derived from peppermint, often presents as a flavor molecule that targets the gustatory system and oropharyngeal thermoreceptors [1]. Research has demonstrated that when MEN is rinsed in the oral cavity it activates the transient receptor potential membrane 8 (TRPM8) [1]. The TRPM8 is a non-selective, temperature-sensitive, and voltage-dependent ion channel predominantly expressed in a subpopulation of thermoceptive/nociceptive neurons found in the dorsal root and trigeminal ganglia [2]. It is suggested that the TRPM8 receptor can be stimulated by either cold temperatures (<22 °C) or by MEN. It is suggested that MEN’s mechanism of action is focused on the reward centers in the brain that appear to trigger several intracellular signaling cascades within the central nervous system (CNS), speculated to attenuate the effects of impaired muscle activation caused by the emergence of exercise induced central fatigue [1,2,3]. What is not known is the combined effect of a MEN mouth rinse (MR) and cold water on the activation of the TRPM8 receptor, downward activation of the CNS, and fatigue perception and physical performance.

Previous research has demonstrated that during moderate to high intensity endurance exercise (>20 min) in a hot environment, a MEN MR can elicit a cooling sensation attenuating the perception of fatigue, leading to increases in ventilatory drive, enabling an individual to work harder to complete an exercise task [2,4,5,6,7]. While it has been documented that MEN has an effect on performance when applied topically and consumed [6], an area gaining great interest is MEN mouth rinsing (MR) [6,8]. Some published research has demonstrated the efficacy of MEN MR at different concentrations, frequencies, and temperatures when used during endurance tasks in hot environments (≥30 °C) [4,6,7,8,9,10]. With oral swilling, MEN appears to lower the thermal sensation and decrease the ratings of perceived exertion (RPE) for a given workload [6,11,12], providing a perceptual “cooling” benefit, which has been shown to improve performance during prolonged exercise in the heat [6,9,10,11].

While MEN MR has been studied during exercise in hot conditions, less is known about the effect MEN MR may have during endurance exercise in a thermoneutral (19–22 °C) environment [8,13,14]. To date, there is only one known study that has investigated the effects of repeated MEN MR on exercise performance in thermoneutral conditions [8,15]. This study demonstrated no performance improvement with the use of a MEN MR on strength and power exercises [15]. The effect of a MEN MR on endurance performance in a thermoneutral environment remains unknown [8]. Moreover, the synergistic effect of MEN MR and fluid temperature is also unknown. While previous research suggests a summation effect when MEN is consumed with a beverage [16], MEN MR has never been explored. Therefore, the purpose of this study was to determine the effect of a MEN MR on cycling TT performance in thermoneutral conditions and to explore the impact of fluid temperature (cold water (CW) or thermoneutral water (TNW)) on menthol’s effect on performance.

We hypothesized that (1) MEN MR would improve the TT performance compared to water alone and (2) that MEN combined with cold water (MEN + CW) would improve TT performance more than the other MR conditions due to a synergistic effect on the “cooling sensation” reducing the thermoperception and RPE and thus improving the TT performance.

## 2. Methods

### 2.1. Participants

Twelve male cyclists (*n* = 12; age, 24.2 ± 3.2 yrs; height, 179.0 ± 6.9 cm; weight, 75.4 ± 12.4 kg) participated in this study. All participants were members of local road cycling clubs, had a minimum of two years of road cycling experience, and participated in cycle-specific training at least three days per week (hours of training per week, 11.3 ± 3.8; *V*O_2 peak_, 61.4 ± 12.1 mL/kg/min; power at *V*O_2 peak_, 370.8 ± 81.8 W). Prior to giving their oral and written informed consent, participants were informed of the experimental protocol and potential risks and were told the purpose of the study was to compare MR temperature preference—this was used as a deception tool to blind the participants from the main purpose of the study. At the completion of the study, all participants were told the true purpose of the study. The study was conducted according to the guidelines of the Declaration of Helsinki and approved by the Institutional Review Board of Ontario Tech University (REB#16074 on 1 October 2020). The participants were asked to keep the same training plan from one trial to the next and to refrain from exercise and caffeine use 24 h before each trial. Testing occurred at the same time each day to reduce the impact of circadian rhythm fluctuation. Each participant was asked to complete a 24 h food log and to repeat the same nutritional intake before each trial—the participants were sent a reminder 48 h prior to each trial.

### 2.2. Study Design

Participants were asked to perform a cycling TT in thermoneutral conditions (~21 °C, 40% RH) with either MEN + TNW, MEN + CW, CW, or TNW MR. The sample of 12 was calculated using G*Power statistical software version 3.1.9.7 (G*Power, Aichach, Germany, 1971) and based on previous research suggesting that MEN reduced the TT completion time by 3 min with an SD of 3.5 min, a standard power of 0.80, and a critical *p* value of 0.05 [11,15,17,18,19].

### 2.3. Preliminary Measurements—VO_2 peak_ Determination

Upon arrival to the laboratory, participants were asked to present their 24 h food log and were weighed in cycling shorts. Participants were then fitted with a respiratory mask for the collection of resting and exercise respiratory gases using a metabolic cart (Parvo TrueOne^®^ 2400 Metabolic Measurement System; ParvoMedics, Inc., Sandy, UT, USA). Participants then completed a 10 min cycling warm-up at a self-selected pace, rested for 3 min, and then completed a maximal incremental cycling test until volitional fatigue on an electromagnetically braked cycle ergometer (Velotron; Racer Mate Inc., Seattle, WA, USA). The incremental cycling test utilized a protocol previously described [11], whereby participants started cycling at 25 Watts (W) and increased in 25 W increments every 1 min until voluntary exhaustion. At the conclusion of each W increment, the rating of perceived exertion 6–20 (RPE) [20] and the heart rate (HR) (Polar H7, Kempele, Finland) were recorded. Respiratory gases were collected and analyzed using a 30 s average VO_2_ from each stage [21]. The *V*O_2 peak_ was defined as the highest 30 s average during the last 30 s of the test. Upon completion of the maximal incremental cycling test, participants cooled down for 5 min at a self-selected pace; then, they were instructed to sit and rest for 30 min with ad libitum fluid intake before completing the familiarization trial.

### 2.4. Familiarization and Experimental Protocol

**Figure 1:**
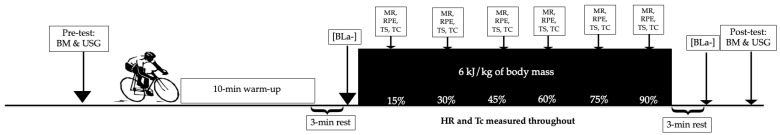
Schematic of the study design. BM, body mass; USG, urine specific gravity; [BLa-], blood lactate; min, minute; MR, mouth rinse; RPE, Ratings of Perceived Exertion; TS, thermal sensation; TC, thermal comfort; HR, heart rate; Tc, core temperature.

Each participant performed one familiarization and four experimental trials, each composed of a cycling time trial, where they were asked to complete 6 kilojoules (kJ)/kg body mass of work in the fastest time possible [22]. The mean laboratory room temperature and relative humidity between the trials was 21 ± 0.2 °C, 40 ± 0.6% relative humidity (Kestral 5400; Nielsen-Kellerman, Boothwyn, PA; accuracy, ± 0.5 °C, ± 2% relative humidity (RH). Following the familiarization trial, the trial order was computer generated in a single-blinded randomized cross-over design and separated by 7 days. Participants were instructed to refrain from exercise 24 h before each trial and repeat their pre-day trial diet. Upon arrival to the laboratory, participants voided their bladder and provided a mid-stream urine sample to determine their hydration status with a portable refractometer (ATAGO USA, Inc., Bellevue, WA, USA). If the participant’s concentration of urine was above 1.020 USG, they were deemed dehydrated [23,24] and instructed to consume 500 mL of water and wait 60 min. If the participant was still deemed dehydrated, they were rescheduled. Participants were then weighed in their cycling shorts, which was repeated post-TT to determine sweat loss. During the trial, no food or water was consumed by the participant. The familiarization trial consisted of a TNW MR, whereas the four randomized experimental trials consisted of the following MR conditions; (1) MEN + TNW, (2) MEN + CW, (3) CW, or (4) TNW. The MR procedure entailed swilling with 25 mL of the MR for 5 s before expectorating into a collection container. The MR occurred a total of 7 times (at rest immediately prior to the TT, 15, 30, 45, 60, 75, and 90% of work completed), with HR, RPE, thermal sensation (TS) [25], and thermal comfort (TC) [26] recorded at each time point (Figure 1). A small capillary blood sample was collected and analyzed for blood lactate concentration (mmol·L^−1^) with an Edge Lactate Analyzer (Red Med, Warszawa, Poland) before and after each time trial. The core body temperature was monitored with a CORE body sensor (greenTag, Zurich, Switzerland) [27]. Sweat loss was determined as follows [22]: sweat loss = (pre-BM − post-BM (kg)) + fluid intake (mL) − urine loss (mL). 

### 2.5. Drink Formulation

The MEN solution was formulated from L-MEN crystals (Sigma-Aldrich; Merck KGaA, Darmstadt, Germany) dissolved in deionized water heated to 40 °C [17]. The solution was stored for a maximum of two weeks at 5 °C. A MEN + TNW was made by warming the MEN solution with a teakettle to >22 °C, and the MEN + CW solution was made by cooling the MEN solution to <5 °C [3].

### 2.6. Statistical Analysis

All analysis was performed in Prism 9 statistical software version 9.3.1 (Graphpad Software, Boston, MA, USA). The time versus condition data were tested using a two-way repeated measures analysis of variance (ANOVA), and Tukey’s post hoc test was used to detect singular differences between conditions. Categorical data were described using the median (IQR). Time versus condition categorical data were tested using the Friedman test; to detect singular differences, a Dunn–Bonferroni test was used. Statistical significance was accepted at *p* < 0.05. Data are presented as means ± standard deviation.

## 3. Results

### 3.1. Trial Conditions

The mean laboratory temperature was 21.2 ± 2.0 °C (20.7–22.0 °C, *p* = 0.923), and the relative humidity was 39.6 ± 0.6% (37–43.9%, *p* = 0.748) for each trial, with no significant difference between the four experimental trials. The mean pre-trial USG was 1.012 ± 0.006 (1.002–1.028) between trials, indicating that the majority of participants were hydrated prior to each experimental trial with no significant difference in the mean USG or individual USG between trials (*p* > 0.05). Participants self-reported sleeping an average of 7.9 ± 1.3 h the night prior to each trial, with no significant difference in the number of hours slept between trials (*p* = 0.783).

### 3.2. Effect of MR on Cycling Time Trial Performance

**Figure 2:**
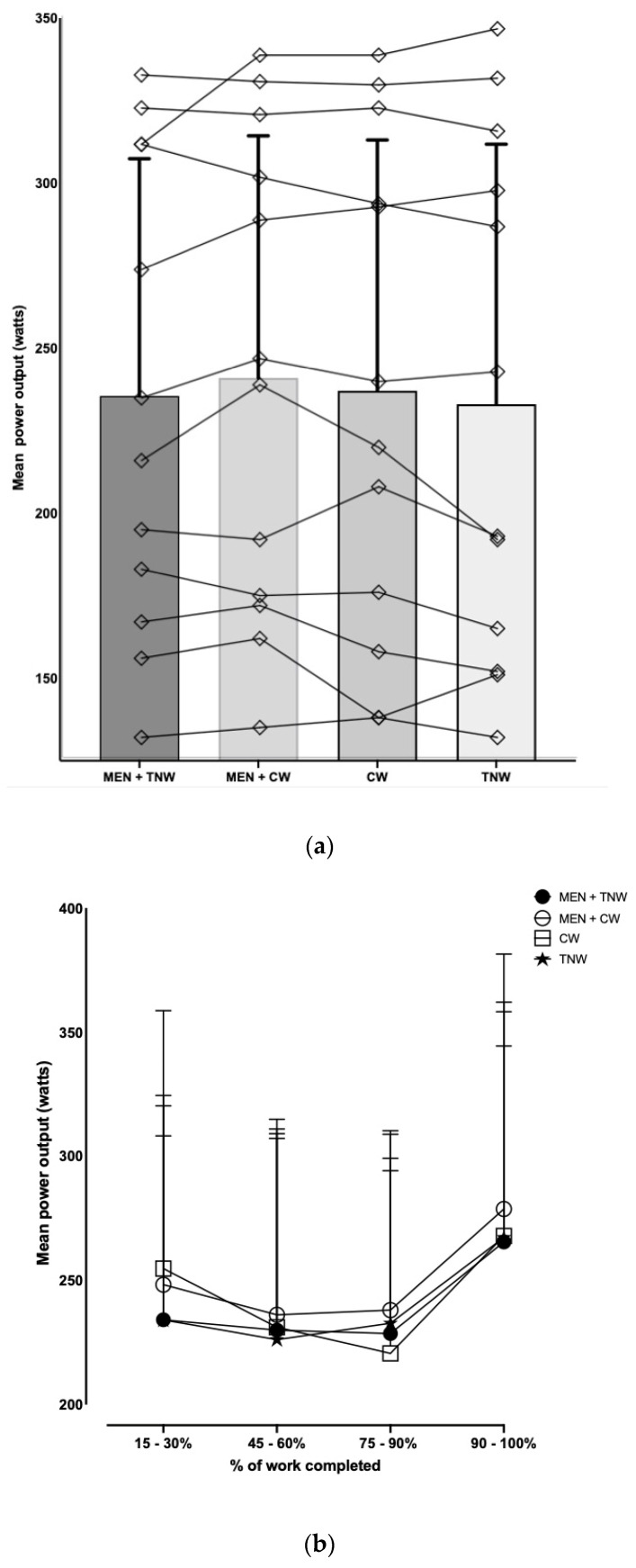
The effect of mouth rinse on (**a**) individual and mean power output in the time trial between trial conditions, and (**b**) mean power output during time trial completion segments (15–30%, 45–60%, 75–90%, and 90–100% completion); Data are mean ± SD (*n* = 12, *p* > 0.05).

There was large individual variability in the time to complete the TT between participants (Figure 2a), with no significant difference found between conditions for each individual (Table 1, *p* = 0.259), but the greatest effect was exhibited in the MEN + CW condition (Table 2). The shortest time to complete the TT was observed for 4/12 participants with MEN + CW, 3/12 participants with MEN + TNW, 3/12 participants with CW, and 2/12 participants with TNW.

The mean power output significantly dropped from the start of the TT to 90% of work completed and then rose significantly from 90 to 100% of work completed as participants anticipated the end of the time trial (Figure 2b, *p* < 0.0001). This was common across time trial conditions, with no significant difference in “pacing strategy” between trial conditions (Figure 2b, *p* > 0.05). There was large individual variability in the mean power output during the TT (131–346 W). The mean power output did not significantly differ between trials (Table 1). There was no significant difference in the mean power output at 15, 30, 45, 60, 75, or 90% of work completed between trials (*p* = 0.691, Figure 2b). The highest mean power output was observed for 4/12 participants with MEN + CW, 4/12 participants with MEN + TNW, 3/12 participants with CW trial, and 1/12 participants with TNW. 

### 3.3. Cardiovascular, Thermoregulatory, and Blood Lactate Response

The heart rate significantly increased throughout the time trial (*p* < 0.0001). There was no significant difference in the mean HR between trials (Table 2). 

The mean sweat loss during the time trial was 0.9 ± 0.4 L (range: 0.5–1.5 L). There was no significant difference in the sweat loss between trial conditions (Table 2, *p* = 0.538).

The Tc significantly increased throughout the time trial (*p* = 0.0007). The mean increase in Tc was 0.17 ± 0.07 °C (0.11–0.24 °C). There was no significant difference in the mean Tc response between trial conditions (Table 3, *p* = 0.526).

There was no significant difference in the blood lactate concentration before (2.0 ± 1.2 mmol·L^−1^) and after (10.6 ± 3.9 mmol·L^−1^) the time trial between the trial conditions (Table 3, *p* = 0.829).

### 3.4. Ratings of Perceived Exertion, Thermal Sensation, and Thermal Comfort

**Figure 3:**
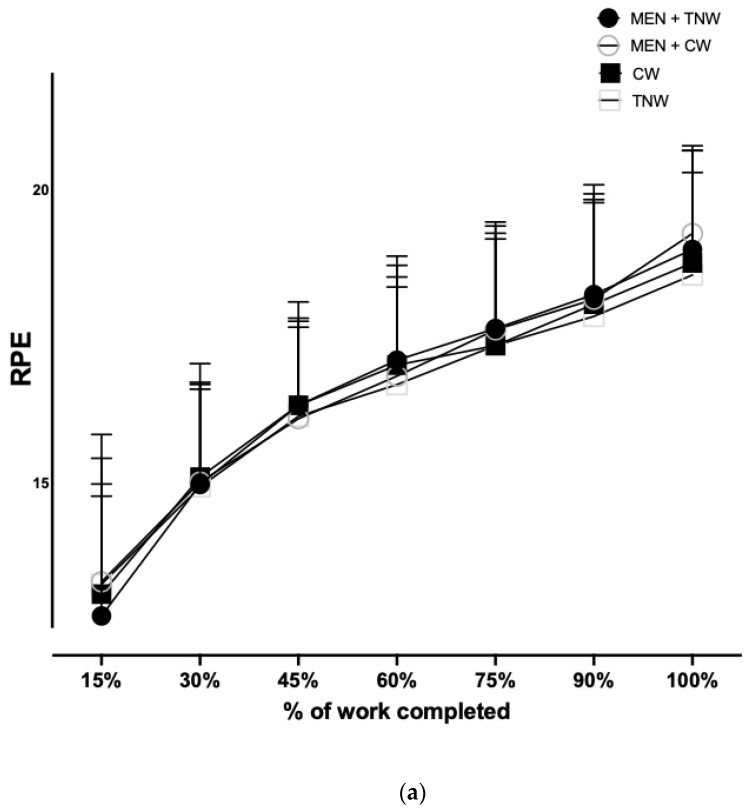

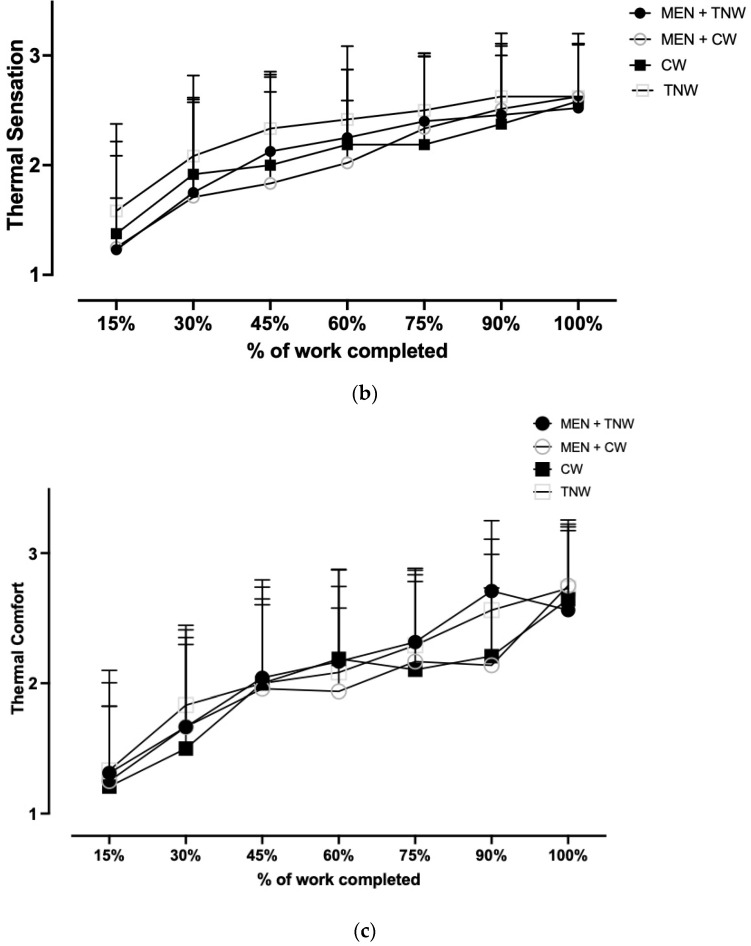
The effects of mouth rinsing (MR) on (**a**) Ratings of Perceived Exertion (RPE), (**b**) thermal sensation (TS), and (**c**) thermal comfort based on the percentage of the time trial completed (%) on cycling TT performance in thermoneutral conditions. Data are mean ± SD (*n* = 12). MR, mouth rinsing; RPE, rating of perceived exertion; TS, thermal sensation; TC, thermal comfort; MEN, menthol; TNW, thermoneutral water; CW, cold water. (*p* > 0.05).

The RPE significantly increased throughout the time trial (*p* < 0.0001, Figure 3a). The mean RPE did not significantly differ between trial conditions at any time point (*p* = 0.451); Moderate effects were observed in the MEN + CW vs. TNW and MEN + TNW vs. TNW trials (Table 4).

The TS significantly increased throughout the time trial (*p* < 0.0001, Figure 3b,c). There was no significant difference in the mean TS (*p* = 0.665). There were small to strong effects observed for MEN + TNW vs. MEN + CW, MEN + CW vs. TNW, and CW vs. TNW (Table 4).

The TC significantly increased throughout the time trial (*p* < 0.0001, Figure 3b,c). There was no significant difference in the mean TC (*p* = 0.538) between trial conditions. Strong effects were observed for MEN + TNW vs. MEN + CW, MEN + TNW vs. CW, and MEN + CW vs. TNW (Table 4).

## 4. Discussion

This is the first known study to investigate the effects of a repeated MEN MR on cycling time trial performance in thermoneutral conditions, while investigating two different MR temperatures. In this study, a MEN MR administered several times throughout exercise did not improve the cycling TT performance compared to a mouth rinse of CW or TNW. Moreover, neither MEN + CW or MEN+TNW significantly improved the cycling power output and perceptual (RPE, TS, and TC) and thermoregulatory responses (HR, sweat loss, and core body temperature), compared to a CW or TNW mouth rinse in thermoneutral conditions. Although MEN MR has shown performance benefits in “hot environments” [4,9,10,11,15,19], these results suggest that MEN MR, combined with or without CW, does not result in a significant difference during endurance exercise in thermoneutral conditions.

### 4.1. Influence of Menthol Mouth Rinsing on Cycling Performance

This study demonstrated that there was not a significant difference in the cycling TT performance with a repeated MEN MR in thermoneutral conditions when MEN was combined with either CW or TNW. Although the results of this study are in contrast to research demonstrating the significant effect of MEN MR on cycling performance in hot conditions [6,11], the results are aligned with the one available paper investigating the effects of MEN MR in thermoneutral conditions [15] and a recent meta-analysis by Gavel et al. [8] reported that there was no significant improvement in the vertical jump, isometric mid-thigh pull, or six-second sprint performance with the use of repeated MEN MR. In both the study by [15] and the current one, it is plausible that the overall stress of the thermoneutral environment and the MR stimulus (despite repeated MEN MR) may not have been strong enough to elicit a central effect leading to increased motor output and an acute increase in exercise performance [3]. Although not statistically significant, our results demonstrate a moderate effect and trend in higher mean power output in the MEN + CW trial from 45% of work completed to the end of the TT (Figure 2); however, with the large variability between participants in power output, the difference is statistically insignificant. This effect and trend align with published work on MEN MR demonstrating improved endurance performance in the latter stages of exercise in hot conditions [6,9,10,11,15,28,29]. Limitations in study methodology, specifically, a lack of tight control in the training status (large SD in mean power output) may have blunted the statistical differences in the mean power output between MR conditions. Future research is required to explore the relationship between MEN MR during exercise in thermoneutral conditions with tighter participant controls on training status, as well as further understanding the frequency/timing of MEN MR to elicit an improvement in performance.

This study does demonstrate the individual differences in responsivity to the use of MEN in thermoneutral conditions. For instance, seven of twelve athletes did have the best TT performance time when using a MEN MR, four athletes with MEN + CW and three athletes with the use of MEN + TNW. Although not statistically significant, marginal improvements with the use of MEN during a cycling TT may be the difference between a podium performance and a mere finishing result. The individual differences in the response to MEN affirms previous recommendations that a ‘one size fits all’ approach to the use of MEN MR is not appropriate; rather, the use of MEN to enhance performance must be considered on an individual basis.

### 4.2. Influence of MR and Fluid Temperature

This study reported no significant effect on cycling performance (physiological and perceptual markers) with a temperature difference of the MEN MR (MEN + CW vs. MEN + TNW). Our results differ from the published work by Tran Trong et al. [16] and Riera et al. [30], who demonstrated that MEN combined with ice or cold water was more beneficial than MEN + thermoneutral water, with the greatest benefit to performance observed in the MEN + ice trial. It must be noted that the aforementioned studies differ in methodology compared to the present study; specifically, participants ingested a MEN drink (not a MR), and the study was completed in a hot environment (28.7 °C ± 0.5 °C, and 30.7 °C ± 0.8 °C, respectively). The contrasting results may be due to the greater surface area affected by the ingestion of a beverage versus an MR and the difference in environmental conditions. More research is needed to determine the most efficacious method of MEN use (MR versus MEN ingestion) in thermoneutral conditions.

### 4.3. Impact of Fluid Temperature on Menthol’s Effect

Research on MEN suggests that a MEN MR can help attenuate fatigue via modulations in brain activity [6,31]. It has been suggested that although certain MRs can increase activity in the reward centers, fluid temperature may impact the severity of the response [3]. In the study by Guest et al. [3], the authors demonstrated that fluid at 50 °C was less pleasant than 20 °C and 5 °C, and this was correlated with greater activity among the primary taste cortex, lateral orbitofrontal cortex, somatosensory cortex, and amygdala, where pleasantness was correlated with activations in the medial orbitofrontal cortex. Our results report no statistically significant performance improvement with MEN + CW suggesting that the stimulus, given the overall heat load of the participant (i.e., environmental conditions, metabolic heat storage, core body temperature, and skin temperature), may not have been strong enough to activate the TRPM8 receptors. However, as noted above, there was a trend for a higher mean power output with MEN + CW from 45% of work completed to the end of the TT, which aligns with the work by Guest et al. [3] linking the severity of the response to the fluid temperature. In addition, previous research has demonstrated that the greatest brain activation in thermoneutral conditions occurred when one MR was contrasted with another—50 °C versus 5 °C. Given that MEN+TNW has consistently shown a performance benefit in “hot conditions” [6], the temperature contrast between the athlete and the MR may be an important consideration linking the mechanism of MEN to the potential ergogenic effect on performance. Further research is needed to explore temperature contrasts (environment vs. MR vs. individual heat load) and modes of MEN use (ingested vs. swilled) to further investigate the effectiveness of MEN MR in thermoneutral environments.

### 4.4. Limitations

Although participant recruitment was kept within a ‘trained’ status (*V*O_2 peak_, 61.4 ± 12.1), there was high inter-participant variability (large SD) in mean power output, which may have blunted the statistical differences seen between trial conditions. Although sleep was tracked, the quality of sleep and sleep debt could have impacted the sensitivity of the MR given its relationship with central fatigue. There was a lack of a defined control group, and the skin temperature was not analyzed.

## 5. Conclusions

This is the first study to observe the effect of a menthol mouth rinse on endurance TT performance in thermoneutral conditions, accounting for fluid temperature. Although the results demonstrate no significant difference in TT performance between MR conditions, there was a trend for improved mean power output in the MEN + CW trial from 45% of work (~16 min) completed onwards. It should be noted that individual responses did vary with 7 of 12 athletes having their best TT performance with the use of a MEN MR, suggesting that MEN use should be considered on an individual basis, as people respond differently.

## Figures and Tables

**Table 1:** Time trial duration (min:sec) and mean power output (Watts (W)) between trial conditions.

	MEN + TNW	MEN + CW	CW	TNW
TT duration (mins:sec)	38:11 ± 12:48	37:21 ± 13:00	38:12 ± 13:54	39:06 ± 14:12
Mean TT PO (W)	236 ± 72	241 ± 73	237 ± 76	233 ± 79
15–30% PO (W)	234 ± 74	248 ± 76	255 ± 104	234 ± 86
45–60% PO (W)	230 ± 77	236 ± 73	231 ± 80	226 ± 89
75–90% PO (W)	229 ± 71	238 ± 72	221 ± 74	233 ± 76
90–100% PO (W)	266 ± 79	279 ± 103	268 ± 94	267 ± 91

Data are mean ± SD (*n* = 12). TT, time trial; mins, minutes; PO, power output; W, watts; MEN, menthol; CW, cold water; TNW, thermoneutral water. There is no significant difference between trial conditions (*p* > 0.05).

**Table 2:** Effect of mouth-swilling conditions for mean time trial time (secs) and mean power throughout trial (Watts).

Variable	Swill	Comparator	Hedge’s g	95% CI
Mean time trial (secs)	MEN + TNW	MEN + CW	g = 0.36	−36.69 to 141.0
	MEN + TNW	CW	g = 0.05	−166.0 to 149.3
	MEN + TNW	TNW	g = 0.4	−214.2 to 90.04
	MEN + CW	CW	g = 0.4	−225.9 to 104.9
	MEN + CW	TNW	g = 0.74	−256.4 to 27.9
	CW	TNW	g = 0.34	−197.9 to 90.41
Mean power output (Watts)	MEN + TNW	MEN + CW	g = 0.4	−15.7 to 4.7
	MEN + TNW	CW	g = 0.11	−13.57 to 10.4
	MEN + TNW	TNW	g = 0.18	−13.3 to 18.3
	MEN + CW	CW	g = 0.28	−5.7 to 13.5
	MEN + CW	TNW	g = 0.57	−5.1 to 21.1
	CW	TNW	g = 0.28	−5.6 to 13.8

CI, confidence interval; g, Hedge’s g.

**Table 3:** Mean heart rate, sweat loss, core temperature, and blood lactate concentration between time trial conditions.

	MEN + TNW	MEN + CW	CW	TNW
Heart Rate (bpm)	175 ± 13	175 ± 5	177 ± 14	172 ± 14
Sweat Loss (L)	0.8 ± 0.2	0.9 ± 0.4	1.0 ± 0.3	1.0 ± 0.5
Mean Tc (°C)	37.8 ± 0.3	37.6 ± 0.3	37.8 ± 0.5	37.8 ± 0.4
△[BLa] (mmol·L^−1^)	9.2 ± 3.3	8.7 ± 4.2	8.3 ± 3.4	7.6 ± 2.0

Data are mean ± SD (*n* = 12). MEN, menthol; TNW, thermoneutral water; CW, cold water; bpm, beats per minute; L, liters; Tc, core body temperature; °C, degrees Celsius; △, delta, change in pre to post blood lactate concentration; [BLa], blood lactate concentration; mmol·L^−1^, millimoles per liter. There is no significant difference between trial conditions (*p* > 0.05).

**Table 4:** Effect of mouth-swilling conditions for mean RPE, TS, and TC in each trial.

Variable	Swill	Comparator	Hedge’s g	95% CI
RPE	MEN + TNW	MEN + CW	g = 0.18	−1.8 to 1.7
	MEN + TNW	CW	g = 0.14	−1.7 to 1.7
	MEN + TNW	TNW	g = 0.55	−1.5 to 1.9
	MEN + CW	CW	g = 0.32	−1.6 to 1.8
	MEN + CW	TNW	g = 0.73	−1.5 to 1.9
	CW	TNW	g = 0.42	−1.6 to 1.8
Thermal Sensation	MEN + TNW	MEN + CW	g = 0.55	−0.6 to 0.7
	MEN + TNW	CW	g = 0.14	−0.6 to 0.6
	MEN + TNW	TNW	g = 2.39	−0.8 to 0.4
	MEN + CW	CW	g = 0.39	−0.7 to 0.6
	MEN + CW	TNW	g = 2.64	−0.9 to 0.4
	CW	TNW	g = 2.19	−0.8 to 0.4
Thermal Comfort	MEN + TNW	MEN + CW	g = 1.29	−0.5 to 0.7
	MEN + TNW	CW	g = 1.22	−0.5 to 0.7
	MEN + TNW	TNW	g = 0.09	−0.6 to 0.6
	MEN + CW	CW	g = 0.02	−0.6 to 0.6
	MEN + CW	TNW	g = 1.37	−0.7 to 0.5
	CW	TNW	g = 1.29	−0.7 to 0.5

CI, confidence interval; g, Hedge’s g.

## Data Availability

The original contributions presented in the study are included in the article, further inquiries can be directed to the corresponding author.
